# Neuraminidase 1 Exacerbating Aortic Dissection by Governing a Pro-Inflammatory Program in Macrophages

**DOI:** 10.3389/fcvm.2021.788645

**Published:** 2021-11-18

**Authors:** Qian Wang, Zhaoyang Chen, Xiaoping Peng, Zeqi Zheng, Aiping Le, Junjie Guo, Leilei Ma, Hongtao Shi, Kang Yao, Shuning Zhang, Zhenzhong Zheng, Jianbing Zhu

**Affiliations:** ^1^Department of Blood Transfusion, The First Affiliated Hospital of Nanchang University, Nanchang, China; ^2^Department of Cardiology, Union Hospital, Fujian Medical University, Fuzhou, China; ^3^Department of Cardiology, The First Affiliated Hospital of Nanchang University, Nanchang, China; ^4^Department of Cardiology, Jiangxi Hypertension Research Institute, Nanchang, China; ^5^Department of Cardiology, Affiliated Hospital of Qingdao University, Qingdao, China; ^6^Department of Cardiology, Shanghai Institute of Cardiovascular Diseases, Zhongshan Hospital, Fudan University, Shanghai, China

**Keywords:** NEU1, aortic dissection, vascular remodeling, macrophage polarization, MMP

## Abstract

Inflammation plays an important role in aortic dissection (AD). Macrophages are critically involved in the inflammation after aortic injury. Neuraminidases (NEUs) are a family of enzymes that catalyze the cleavage of terminal sialic acids from glycoproteins or glycolipids, which is emerging as a regulator of macrophage-associated immune responses. However, the role of neuraminidase 1 (NEU1) in pathological vascular remodeling of AD remains largely unknown. This study sought to characterize the role and identify the potential mechanism of NEU1 in pathological aortic degeneration. After β-aminopropionitrile monofumarate (BAPN) administration, NEU1 elevated significantly in the lesion zone of the aorta. Global or macrophage-specific NEU1 knockout (NEU1 CKO) mice had no baseline aortic defects but manifested improved aorta function, and decreased mortality due to aortic rupture. Improved outcomes in NEU1 CKO mice subjected to BAPN treatment were associated with the ameliorated vascular inflammation, lowered apoptosis, decreased reactive oxygen species production, mitigated extracellular matrix degradation, and improved M2 macrophage polarization. Furthermore, macrophages sorted from the aorta of NEU1 CKO mice displayed a significant increase of M2 macrophage markers and a marked decrease of M1 macrophage markers compared with the controls. To summarize, the present study demonstrated that macrophage-derived NEU1 is critical for vascular homeostasis. NEU1 exacerbates BAPN-induced pathological vascular remodeling. NEU1 may therefore represent a potential therapeutic target for the treatment of AD.

## Introduction

Aortic dissection (AD) is a fatal surgical emergency characterized by acute-onset chest or back pain with few, if any, preceding signs (1). The mortality rate is 60–70% in the first 24 h (2). Anatomically, when the intima of the aorta is damaged and ruptured for various reasons, the flow of blood through the tears and separated the media into two layers, leading to the further destruction of the aortic wall (3). Despite new concepts regarding the diagnosis, classification, and treatment of AD have been developed recently (4), little is known about the pathological and molecular mechanisms before and after the onset of AD due to its sudden and unpredictable nature (5). Therefore, ongoing research is emergency for elucidating the pathophysiology of AD and developing diagnostic and therapeutic intervention methods.

The pathophysiology of AD has gradually been elucidated. In addition to mutations in some genes involved in extracellular matrix metabolism and smooth muscle cytoskeleton, some high-risk factors for AD have also been confirmed, including long-term hypertension, dyslipidemia, smoking, giant cell arteritis, etc. These non-genetic factors suggest that the inflammation may make the aorta susceptible to AD (6). Inflammatory cells infiltrate the injured site of the aorta to remove necrotic cells and damaged tissue; however, the excessive inflammation may play a role in aneurysm formation after dissection (7). Although the degeneration of the media is a fundamental pathological change of AD, the degeneration only weakens the media, and most cases of AD form intimal tears at the beginning. Further studies have shown that the inflammation is an important mechanism leading to intimal damage and mid-layer degeneration (8). Moreover, an analysis of gene expression changes in human dissecting tissues using cDNA microarrays confirmed that the inflammation was involved in the pathogenesis of disease (9). Indeed, numerous studies confirmed that inflammatory cytokines and chemokines, including interleukin (IL)-6, granulocyte colony-stimulating factor, granulocyte macrophage colony-stimulating factor, IL-17, chemokine (C–X–C motif) ligand 1, and C–C motif chemokine ligand 2, play essential roles in AD pathogenesis (10–12). IL-6-STAT3 signaling pathway promotes AD induced by angiotensin (Ang) II *via* the Th17/IL-17 axis in mice (13).

Different types of immune cells infiltrated in the lesion area of the AD aorta, among which macrophages are the most abundant cell type (14). Evidence from animal models and patients showed marked infiltration of macrophages at the site of tears (15). Animal model studies revealed the importance of signaling amplification loops between macrophages and fibroblasts *via* IL-6 and monocyte/macrophage chemokine (MCP-1) in AD tissues (10). Macrophage-associated cytokine signaling may be the targets to prevent the development and progression of AD. For example, Socs3 in macrophages modulates the stress response of macrophages and vascular smooth muscle cells (VSMCs) and promotes the healing of damaged aortic walls and preventing AD development in mice, whereas macrophage Socs3 knockout mice showed premature activation of cell proliferation, increased inflammatory response, and the conversion of macrophages to a pro-inflammatory phenotype (5).

Neuraminidases (NEUs), also known as sialidases, are a family of enzymes that cleave sialic acid on the surfaces of cells. NEU1 is the most abundant and ubiquitous of the four mammalian sialidases with a wide tissue distribution (16). In addition to participating in catabolism of glycoproteins and glycolipids *in vivo*, an increasing body of literature suggests that NEU1 also plays an important role in the immune system, especially in the macrophage-related inflammation (17). For example, animal studies have found that sialidase deficiency leads to reduced macrophage effect, whereas the upregulation of NEU1 expression during the differentiation of monocytes into macrophages helps to enhance the phagocytosis of these cells (18). Recently, a study has shown that NEU1 regulates the activation of TLR receptors on macrophages, to be specific, binding of the ligand to TLR induces NEU1 activity, leading to the desalivation of the receptor, which in turn induces receptor activation, nitric oxide, and pro-inflammatory cytokine production (19). Given inflammatory macrophage triggering AD and NEU1 governing macrophage polarization, whether NEU1 is involved in the pathogenesis of AD through the regulation of macrophages has not been reported.

In the present study, we observed that the NEU1 expression was markedly upregulated in aortic tissues from β-aminopropionitrile monofumarate (BAPN)-induced AD mice. Deletion of NEU1 (either global or specifically in macrophage) all manifested improved aorta function, vascular remodeling, and decreased mortality due to aortic rupture. Mechanically, improved outcomes in NEU1 CKO mice were associated with the improved vascular inflammation, which at least in part by promoting the polarization of M2 macrophages. Therefore, it is proposed that NEU1 may be a potential therapeutic target for AD.

## Methods

### Animals

NEU1^F/F^ mice, LysM^Cre^, and NEU1 KO mice (C57BL/6) were purchased from Shanghai Biomodel Organism Co, Shanghai, China. NEU1 gene is comprised of five coding exons 2–6. To generate NEU1^F/F^ mice, a donor vector containing exon 2 flanked by two loxP sites and two homology arms were used as the homologous recombination mediated repairing template. NEU1^F/F^ mice were hybridized with LysM^Cre^ mice to generate NEU1^F/F^, LysM^Cre^ (NEU1 CKO) mice. All experiment animals were male considering the feature of less sex hormone variations and high incidence of thoracic aortic dissection (TAD). Age/weight-matched wild-type (WT) mice served as controls. NEU1 KO, NEU1^F/F^ mice, and NEU1 CKO mice aged 4 weeks were administrated with BAPN in the drinking water with 1 g/kg/day for 4 weeks to induce TAD. All mice shared standard chow and water and were maintained with an alternating 12-h light/dark cycle. All animal procedures were performed in accordance with the protocols approved by the Animal Care and Use Committee of The First Affiliated Hospital of Nanchang University.

### Immunofluorescence Staining

Vascular tissue was firstly fixed with 4% paraformaldehyde (PFA) at room temperature for 2 h. After phosphate-buffered saline (PBS) washing, they were dehydrated with 30% sucrose overnight and embedded in optimum cutting temperature (OCT) solution (Sakura Finetek Inc., Torrance, CA, USA). Before the experiment, the frozen sections (7 μm) were dried at room temperature and fixed in cold acetone solution for 10 min. Sections were sealed with 3% bovine serum albumin (BSA) for 2 h to prevent non-specific binding. COL1A1 (1:200; NBP1-30054; Novus, St. Louis, MO, USA), CD68 (1:200, AB283654; Abcam, Cambridge, UK), inducible nitric oxide synthase (iNOS) (1:200, AB178945; Abcam, Cambridge, UK), arginase-1 (Arg-1) (1:200, 93668; Cell Signaling Technology, Danvers, MA, USA), and NEU1 (1:500, sc-166824; Santa Cruz Biotechnology, Santa Cruz, CA, USA) were incubated at 4°C overnight. An appropriate secondary antibody (1:500) was taken and incubated at room temperature for 2 h. The 4′,6-diamidino-2-phenylindole (DAPI) staining was performed after full rinse-washing. Finally, it was sealed with anti-fade reagent and observed by laser scanning confocal microscope (Carl Zeiss, Oberkochen, Germany). For cell immunofluorescence test, sorted macrophages were cultured on a coverslip placed in 12-well plate. Before the experiment, the slides were fixed with 4% PFA for 30 min, rinsed with PBS, and remaining procedures were according to the frozen sections. The primary antibody used was as follows: iNOS (1:200, AB178945, Abcam, Cambridge, UK) and Arg-1 (1:200, 93668; Cell Signaling Technology, Danvers, MA, USA).

### Dihydroethidium Staining

Dihydroethidium (DHE) assay kit (S0063, Beyotime, Shanghai, China) was used to measure superoxide anions in aorta sections of NEU1^F/F^ and NEU1 CKO mice. Each group of sections was exposed to DHE (10 μM) for 30 min at 37°C. After washing with PBS three times, samples were visualized by confocal microscopy (Olympus Corp., Tokyo, Japan).

### Hematoxylin and Eosin Staining

Hematoxylin and Eosin Kit (ab245880, Abcam, Cambridge, UK) was used for the pathology study of the aorta. Briefly, sections were deparaffinized and hydrated in distilled water. Then, sections were placed in adequate Mayer's Hematoxylin (Lillie's Modification) to completely cover tissue section and incubated for 5 min. Furthermore, slides were rinsed in two changes of distilled water to remove excess stain. Later, slides were applied with adequate bluing reagent to completely cover tissue section and incubated for 10–15 s. Then slides were rinsed in two changes of distilled water and dipped in absolute alcohol and blot off the excess, applied with adequate Eosin Y Solution (Modified Alcoholic) to completely cover tissue section to excess and incubated for 2–3 min. Furthermore, slides were rinsed using absolute alcohol and dehydrated in three changes of absolute alcohol. Clear slides were mounted in synthetic resin.

### Van Gieson's Staining

Van Gieson's Staining Kit (ab150667, Abcam, Cambridge, UK) was used for the study of the elastin degradation. Briefly, sections were deparaffinized and hydrated in distilled water. Then slides were placed in Elastic Stain Solution for 15 min, rinsed in running tap water until no excess stain remains on slides, and were dipped in differentiating solution 15–20 times and rinsed in tap water. Slides were checked microscopically for proper differentiation, rinsed in running tap water, placed in sodium thiosulfate solution for 1 min, and rinsed in running tap water. Stained slides were placed in Van Gieson's solution (ab150667, Abcam, Cambridge, UK) for 2–5 min, rinsed in two changes of 95% alcohol, and dehydrated in absolute alcohol. Clear slides were mounted in synthetic resin.

### TUNEL

Cell Death Detection Kit (C1088, Beyotime, Shanghai, China) was used for cell apoptosis studies. The TUNEL staining was performed according to the instructions of the manufacturer. First, the frozen sections of vascular tissue were fixed with 4% PFA for 30 min and incubated with 0.5% Triton X-100 at room temperature for 5 min. The TUNEL detection solution was added and incubated at 37°C for 60 min in dark. The tissue sections were observed by confocal microscopy. For each aortic treatment, images were captured from three randomly selected views. The number of positive cells and the total number of nuclei in each image were quantitatively analyzed.

### RNA Extraction and Quantitative Real-Time PCR

Total RNA was extracted from aortic tissue using the TRIzol reagent (Invitrogen, Carlsbad, CA, USA), and RNA was isolated from sorting macrophages using the RNeasy Plus Micro Kit (74034, QIAGEN, Hilden, Germany), according to the instructions of the manufacturer. RNA samples (1 μg) were subsequently reverse-transcribed into cDNA with a reverse transcription reagent kit (RR036A, Takara Bio Inc., Kusatsu, Japan), and the resulting cDNA was amplified by RT-PCR using the SYBR Green Mix (11201ES08; Yeasen, Shanghai, China). Each sample was analyzed in triplicate and normalized to a reference RNA. Relative expression levels were quantitated using the ΔΔCt method.

### Western Blotting

The protein concentrations of aortic tissue lysates were determined using a Pierce BCA Protein Assay Kit (Pierce Biotechnology, Waltham, MA, USA). Equal amounts of samples were loaded and separated on 10% SDS-PAGE. Then, the proteins were transferred to nitrocellulose membranes, incubated with 5% skimmed milk for 2 h at room temperature, and incubated with primary antibodies overnight at 4°C. Primary antibodies were used as follows: NEU1 (1:500, sc-166824, Santa Cruz Biotechnology, Santa Cruz, CA, USA) and GAPDH (1:1000, 5174s, Cell Signaling Technology, Danvers, MA, USA) Then, the membranes were incubated with the corresponding secondary antibodies. The blots were visualized using a chemiluminescence reagent. Densitometric analysis for each band was performed using the ImageJ software (National Institutes of Health, Bethesda, MD, USA).

### Flow Cytometry

Macrophages were analyzed and sorted using a fluorescence-activated cell sorter (FACS) (BD Biosciences, San Jose, CA, USA) as previously described. Briefly, the AD tissues were dissected, carefully cut into small pieces, and enzymatically digested with collagenase II (1.5 mg/ml), elastase (0.25 mg/ml), and DNase I (0.5 mg/ml) for 1 h at 37°C. After digestion, the tissues were passed through 70-μm cell strainers. After washing, anti-CD16/32 antibody was used to block the non-specific binding. Fixable viability stain 510 (564406, BD Biosciences, San Jose, CA, USA) and the following antibodies were used for flow cytometry: CD45-APC-Cy7 (557659, BD Biosciences), CD11b-PE-Cy7 (552850, BD Biosciences), F4/80-BV421 (565411, BD Biosciences), CD86-PE (553692, BD Biosciences), and CD206-APC (565250, BD Biosciences). For flow cytometric sorting, cells were resuspended in the FACS buffer at 20 × 10^6^ cells/ ml and separated on a MoFlo High-Performance Cell Sorter (Dako Cytomation, Carpinteria, CA, USA). The results were expressed as the absolute number of cells per mg of tissue. Data were analyzed with the FlowJo software (FlowJo LLC, Ashland, OR, USA).

### Statistical Analysis

All data are presented as the mean ± SEM and analyzed in GraphPad Prism 8.0 (GraphPad Software, San Diego, CA). Data normality was determined by the Shapiro–Wilk test. The significant differences between different data were calculated by unpaired two-tailed *t*-test (for two groups) and one-way or two-way ANOVA (for more than two groups) followed by the Tukey's and Dunnett's multiple comparisons test. The *p*-value lower than 0.05 were considered statistically significant.

## Results

### NEU1 Expression Increases in Aortic Dissecting Tissues

**Figure 1:**
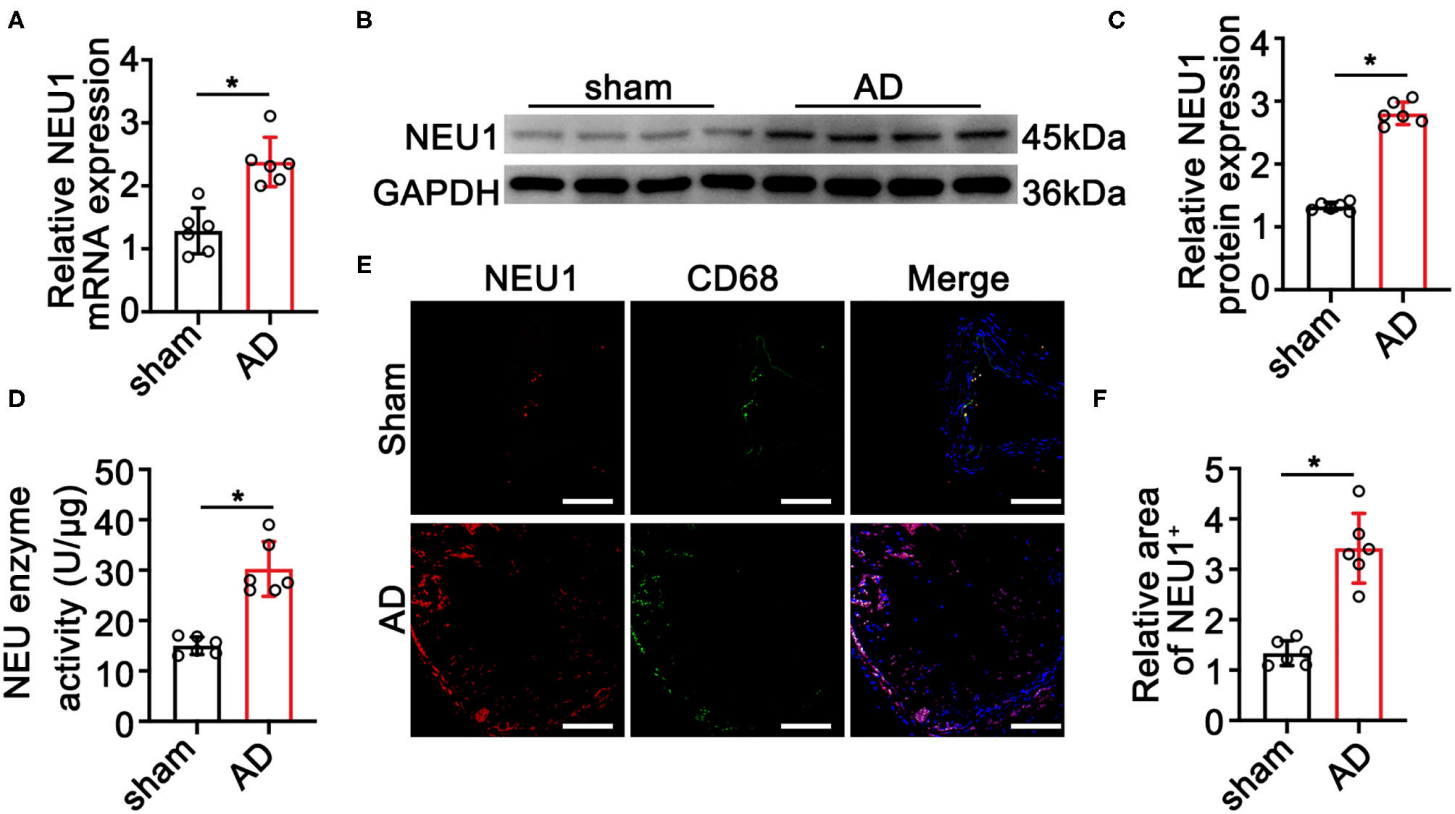
Neuraminidase 1 (NEU1) expression increases in aortic dissecting (AD) tissues. **(A)** Quantitative results of NEU1 mRNA level in aortas of mice subjected to BAPN induction for 4 weeks (*n* = 6 mice per group). **(B,C)** Representative western blots and quantitative results of NEU1 level in aortas of mice subjected to BAPN induction for 4 weeks (*n* = 6 mice per group). **(D)** The neuraminidase enzyme activity of AD in mice by ELISA (*n* = 6 aortas per group). **(E,F)** Immunofluorescence staining with anti-NEU1 (red) and anti-CD68 (green) antibody in slices from the indicated mice aortas (*n* = 6 mice aortas per group; scale bar: 50 μm). Data are presented as mean ± SEM. **(A,C,D,F)** unpaired two-tailed *t*-test. BAPN, β-aminopropionitrile monofumarate; NEU1, neuraminidase 1. ^*^p < 0.05.

To elucidate the role of NEU1 in AD development, we detected the expression of NEU1 in AD tissues. As shown in Figures 1A–C, the mRNA and protein levels of NEU1 were dramatically higher in AD vessels than in normal controls. The NEU1 activity was also elevated in dissecting aortas compared with the controls (Figure 1D). Since NEU1 was reported to highly expressed in macrophages in atherosclerosis vessels (20), we therefore speculated that NEU1 may also highly expressed in macrophages in AD vessels. CD68 and NEU1 immunofluorescence staining revealed NEU1 was mainly located in CD68^+^ macrophages in dissecting aortas (Figures 1E,F). Collectively, these results revealed that NEU1 may play a role in AD development.

### Global NEU1 Deletion Mitigates BAPN-Induced AD Development

**Figure 2:**
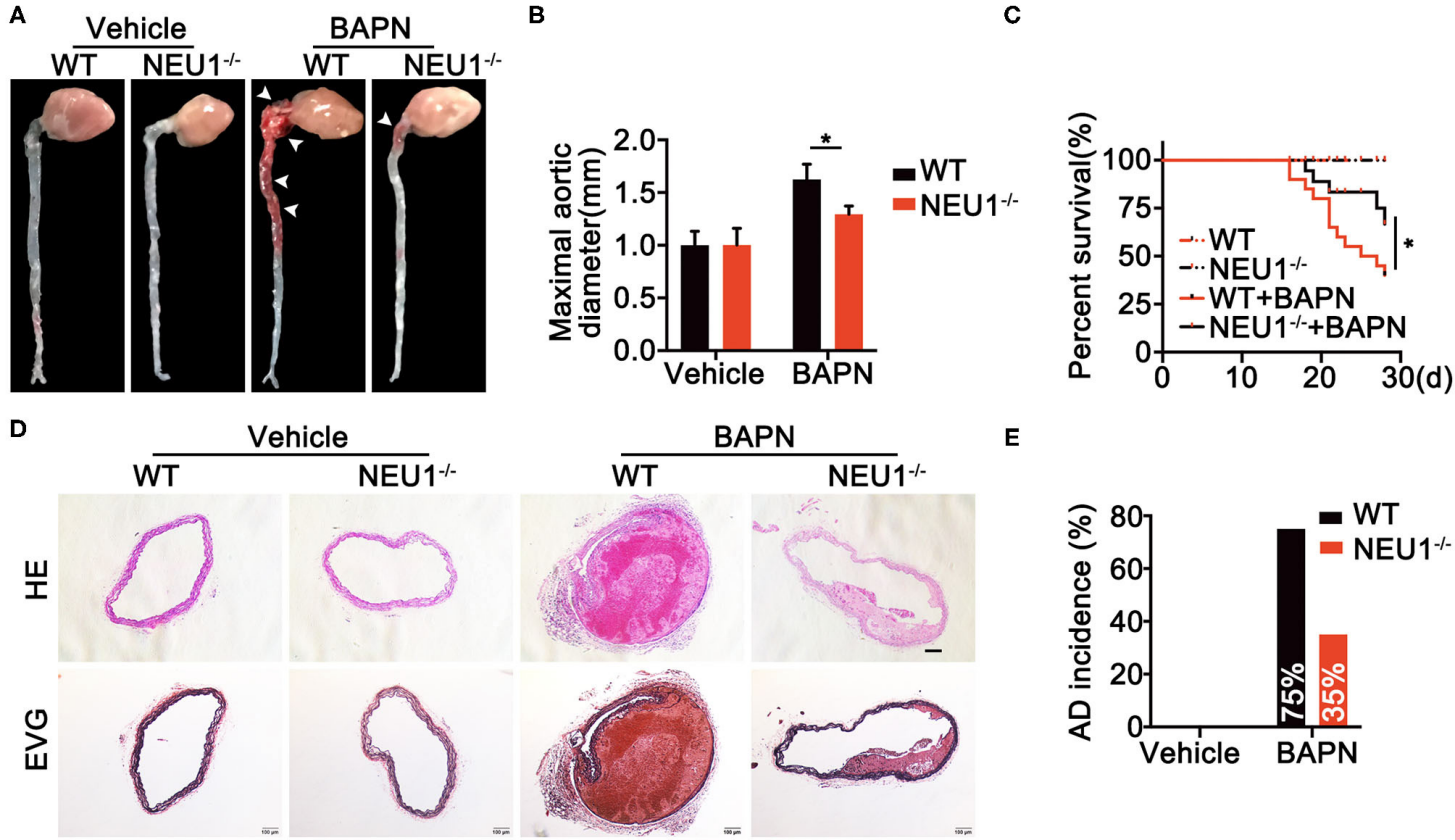
Global NEU1 deletion mitigates BAPN-induced AD development. **(A–E)**, C57BL/6 wild-type and NEU1 KO mice were subjected to BAPN induction. **(A)** Representative macrographs of the aortas. **(B)** Maximal aortic diameter (*n* = 8, *p < 0.05). **(C)** Kaplan–Meier survival analysis of indicated groups (*n* = 20, *p < 0.05). **(D)** Representative HE and EVG staining of the aorta. **(E)** AD incidence in indicated groups (*n* = 20). AD, aortic dissecting; BAPN, β-aminopropionitrile monofumarate; EVG, Elastic Van Gieson's staining; HE, hematoxylin and eosin; KO, knockout; NEU1, neuraminidase 1.

To further discern the role of NEU1 in AD development, a murine AD model was established using 4-week BAPN drinking in C57BL/6-WT and NEU1 KO mice (Supplementary Figure 1A). Interestingly, BAPN administration prompted AD formation, increased maximal aortic diameters, and provoked a remarkable mortality (Figures 2A–C), the effects of which were greatly attenuated by NEU1 KO, with little effect at base line (Figures 2A–C). Moreover, hematoxylin and Verhoeff–van Gieson staining demonstrated that NEU1 deletion mitigated BAPN-induced dissecting aneurysm formation and elastic fiber degradation (Figure 2D). Additionally, AD incidence was also alleviated in NEU1 KO mice (35%) compared with WT controls (75%) (Figure 2E). These findings denoted the benefit of NEU1 deficiency in AD development.

### Macrophage-Specific NEU1 Deletion Mitigates BAPN-Induced AD Development

**Figure 3:**
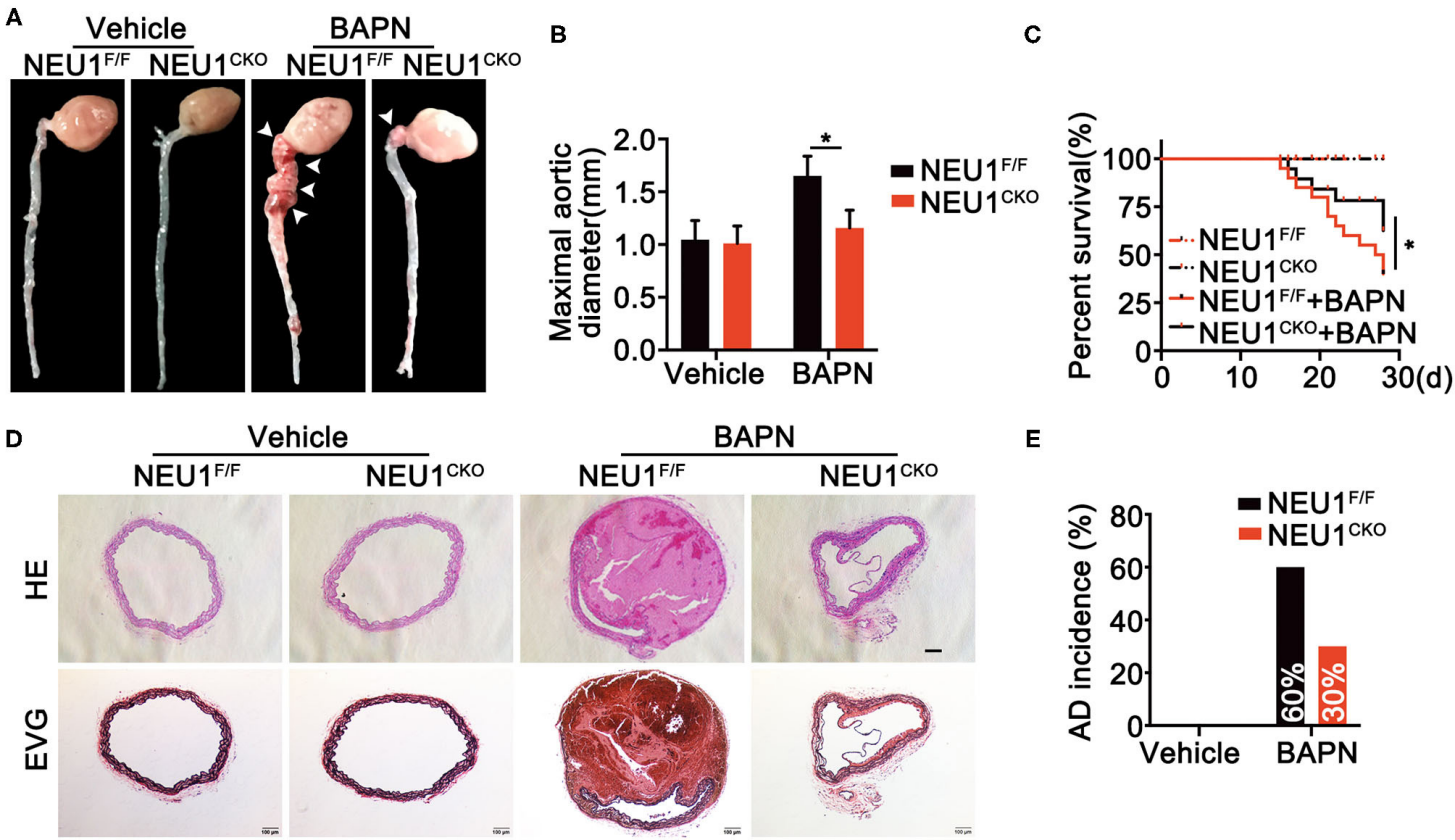
Macrophage-specific NEU1 knockout (NEU1 CKO) deficiency mitigates BAPN-induced AD development. **(A–E)**, NEU1^F/F^ and NEU1 CKO mice were subjected to BAPN induction. **(A)** Representative macrographs of the aortas. **(B)** Maximal aortic diameter (*n* = 9, *p < 0.05). **(C)** Kaplan–Meier survival analysis of indicated groups (*n* = 20, *p < 0.05). **(D)** Representative HE and EVG staining of the aorta. **(E)** AD incidence in indicated groups (*n* = 20). AD, aortic dissecting; BAPN, β-aminopropionitrile monofumarate; HE, hematoxylin and eosin; KO, knockout; NEU1, neuraminidase 1.

Since NEU1 was identified to highly localized in macrophages in lesion area of vessels as shown in Figure 1E, we therefore utilized macrophage NEU1 KO mice (Supplementary Figure 1B) to establish AD model. Coincidently, BAPN administration prompted AD formation, increased maximal aortic diameters, and provoked a remarkable mortality (Figures 3A–C), the effects of which were greatly attenuated by Mac-NEU1 KO (Figures 3A–C). Moreover, hematoxylin and Verhoeff–van Gieson staining demonstrated that macrophage NEU1 deletion mitigated BAPN-induced dissecting aneurysm formation and elastic fiber degradation (Figure 3D). Additionally, AD incidence was also decreased in NEU1 CKO mice (30%) compared with controls (60%) (Figure 3E). These findings denoted the benefit of macrophage NEU1 deficiency in AD development.

### NEU1 Depletion Suppresses BAPN-Induced Inflammation

**Figure 4:**
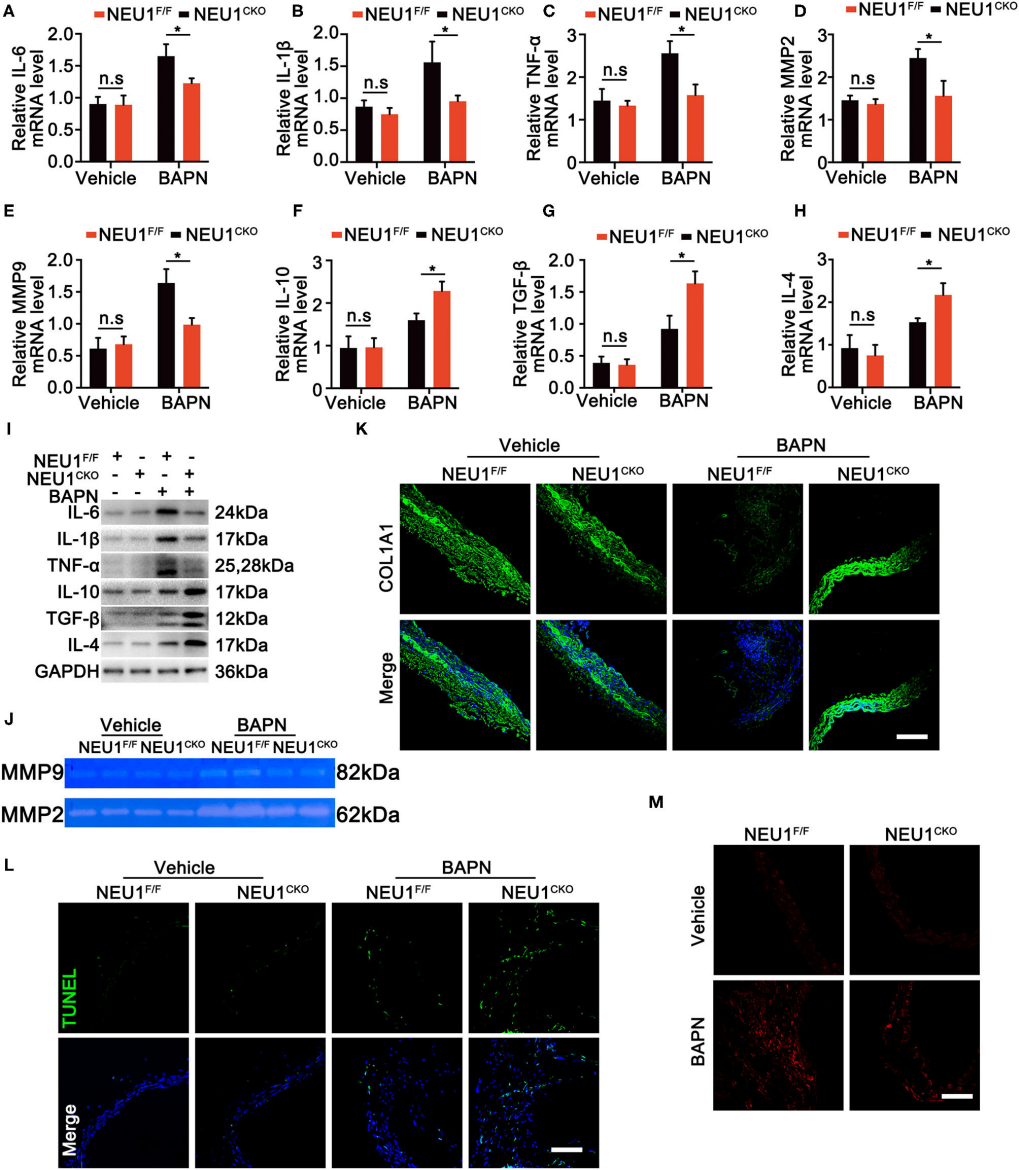
NEU1 depletion suppressed BAPN-induced inflammation. **(A–H)** Quantitative mRNA levels of pro- and anti-inflammatory factors in aortas of NEU1^F/F^ and NEU1 CKO mice subjected to BAPN induction for 4 weeks (*n* = 6 per group). **(I)** Representative western blots of pro- and anti-inflammatory factors in aortas of NEU1^F/F^ and NEU1 CKO mice subjected to BAPN induction for 4 weeks (*n* = 6 per group). **(J)** Gelatin zymogram analysis of MMP2 and MMP9 activity levels in aortas of NEU1^F/F^ and NEU1 CKO mice subjected to BAPN induction for 4 weeks. **(K)** Immunofluorescence images of COL1A1 in aortas of NEU1^F/F^ and NEU1 CKO mice subjected to BAPN induction for 4 weeks (scale bar: 50 μm). **(L)** TUNEL staining of aortas from NEU1^F/F^ and NEU1 CKO mice subjected to BAPN induction (scale bar: 50 μm). **(M)** DHE staining of aortas from NEU1^F/F^ and NEU1 CKO mice subjected to BAPN induction for 4 weeks (scale bar: 50 μm). BAPN, β-aminopropionitrile monofumarate; DHE, dihydroethidium; KO, knockout; MMP, matrix metalloproteinase; NEU1, neuraminidase 1; NEU1 CKO, macrophage-specific NEU1 knockout. *p < 0.05.

Given the role of NEU1 in the inflammation and the localization of NEU1 in macrophages, we therefore detected both pro- and anti-inflammatory factors. Protein and mRNA analysis revealed that BAPN administration enhanced the expression of pro-inflammatory factors like IL-6, IL-1β, tumor necrosis factor (TNF)-α, matrix metalloproteinase (MMP)-2, and MMP-9 in NEU1^F/F^ mice, whereas Mac-NEU1 KO partly reversed the inflammatory state (Figures 4A–E,I). Meanwhile, the expression of anti-inflammatory factors like IL-10, IL-4, and transforming growth factor (TGF)-β was higher in NEU1 CKO mice than those in NEU1^F/F^ mice following BAPN treatment (Figures 4F–H,I). Moreover, the exacerbated cell apoptosis, extracellular matrix (ECM) degradation, MMP 2/9 activity, and reactive oxygen species (ROS) production by BAPN administration were rescued by NEU1 deletion in macrophage (Figures 4J–M). NEU1 deficiency alone affects little in aortic function (Figures 4A–M). The above results demonstrated that NEU1 promoted the inflammation, apoptosis, and ROS production, thus leading to the progression of AD.

### NEU1 Deletion Promotes the M2 Polarization of Macrophages

**Figure 5:**
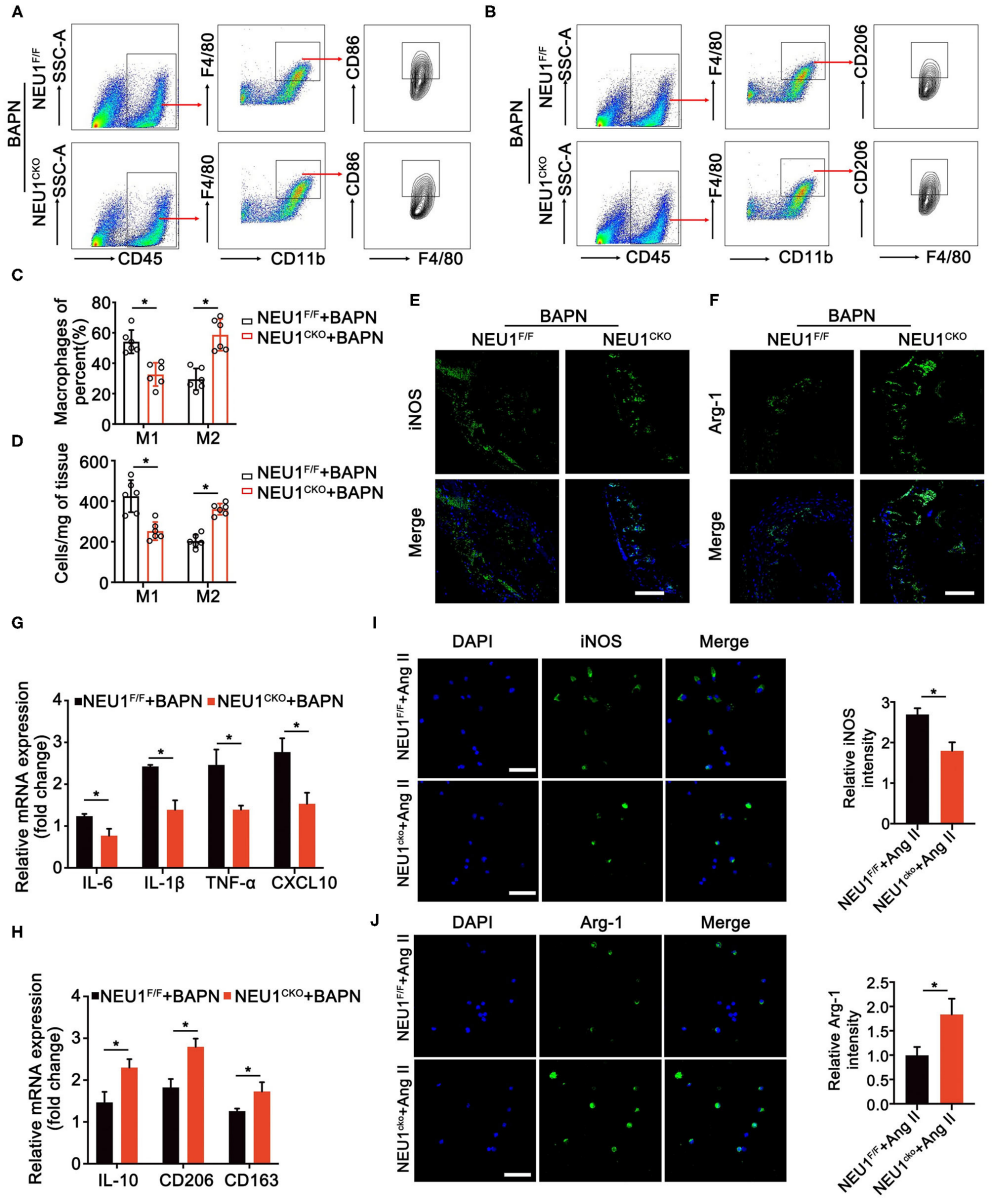
NEU1 deletion promotes the polarization of macrophages. **(A,B)** Gating strategy for CD45^+^CD11b^+^F4/80^+^ CD86^+^ M1 macrophages and CD45^+^CD11b^+^ F4/80^+^ CD206^+^ M2 macrophages in aortas of NEU1 CKO mice and their controls. **(C,D)** Quantification of the CD86^+^ and CD206^+^ macrophages. **(E,F)** Immunofluorescence images of iNOS^+^ and Arg-1^+^ macrophages in aortas of NEU1^F/F^ and NEU1 CKO mice subjected to BAPN induction for 4 weeks (scale bar: 50 μm). **(G,H)** mRNA levels of M1 and M2 markers in macrophages sorted by flow cytometry of NEU1^F/F^ and NEU1 CKO mice subjected to BAPN induction for 4 weeks (*n* = 6, *p < 0.05). **(I,J)** Macrophages sorted from NEU1^F/F^ and NEU1 CKO mice, and subjected to Ang II treatment for 24 h. Immunofluorescence images of iNOS^+^ and Arg-1^+^ macrophages by Ang II treatment (scale bar: 50 μm). Ang, angiotensin; Arg-1, arginase-1; BAPN, β-aminopropionitrile monofumarate; DHE, dihydroethidium; iNOS, inducible nitric oxide synthase; KO, knockout; NEU1, neuraminidase 1; NEU1 CKO, macrophage-specific NEU1 knockout.

Neuraminidase 1 was realized to regulate the inflammation of aorta; we therefore studied the role of NEU1 in macrophage polarization. Flow cytometry analysis of the BAPN-induced aorta revealed that NEU1 deletion promoted the polarization of macrophage from pro-inflammatory to anti-inflammatory state (Figures 5A–D). Immunofluorescence staining of the aorta also displayed that the pro-inflammatory marker iNOS was decreased, and the anti-inflammatory marker Arg-1 was increased by NEU1 depletion in BAPN-induced mice (Figures 5E,F). The mRNA analysis of the macrophages sorted by flow cytometry was coincidence with the previous results (Figures 5G,H). Furthermore, we sorted the macrophages from WT and NEU1 CKO mice and subjected to Ang II treatment for 24 h. Immunofluorescence staining revealed that NEU1-deficient macrophages were prone to M2 polarization (Figures 5I,J). Our findings demonstrated that the critical role of NEU1 in macrophage polarization.

## Discussion

In this study, we found that NEU1 expression was significantly upregulated in dissecting tissues from BAPN-induced AD mice. Additionally, using genetically KO mice, we demonstrated that NEU1 played an important role in the development of AD. Furthermore, the elevated NEU1 expression in macrophages promotes the M1 macrophage polarization. Therefore, NEU1 may become a potential therapeutic target for AD.

Neuraminidases are a family of four different enzymes, NEU1, NEU2, NEU3, and NEU4, which remove the terminal sialic acids from glycoproteins or glycolipids (21). Among the four sialidases, the lysosomal NEU1 has been shown to assume a vital role in immune cells (22). The immune inflammatory response plays an important role in the development of AD. However, whether NEU1 could affect the pathogenesis of AD remains unknown. Previous studies suggest that NEU1 is closely associated with the progression of several cardiovascular diseases. Lipopolysaccharide (LPS), NEU1, and IL-1β act in a positive feedback loop as enhancers of inflammation in monocytes/macrophages and may therefore promote atherosclerosis and plaque instability (20). In addition, upregulation of NEU1 after ischemia/reperfusion (I/R) promotes heart failure by promoting monocyte/macrophage inflammation and enhancing myocardial hypertrophy (23). Based on the above evidence, we speculated whether NEU1 could also participated in the pathogenesis of AD by regulating macrophages.

Considering the elevated NEU1 expression in the aortic tissues of AD mice, we hypothesized the involvement of NEU1 in AD development. Our studies proved that NEU1 deficiency mitigated the AD. Recent investigations provide evidence that NEU1 was mainly expressed in macrophages (20), which is consistent with our immunofluorescence findings. We further obtained NEU1 CKO mice by hybridization of NEU1^F/F^ mice with LysM^cre^ mice to explore the effect of macrophage-derived NEU1 on AD. NEU1 CKO mice also showed significant improvement in aortic injury, consistent with the results of global NEU1 KO mice. Collectively, these observations suggested elevated NEU1 expression in dissecting tissues (especially in macrophages) contributed to aortic vascular remodeling.

Inflammation, apoptosis, ECM degradation, and oxidative stress are responsible for the pathogenesis of AD (24). In our study, we found that the pro-inflammatory factors were significantly decreased in the aortic tissues of NEU1 CKO mice. The dysfunction of VSMCs, including the imbalance between proliferation and apoptosis, is deemed to promote the vascular remodeling (25, 26). We also observed the decreased apoptosis of VSMCs compared with the controls. In addition, VSMCs are the main source of ECM proteins (24, 27). *In vivo* results showed that NEU1 CKO mice significantly reduced collagen 1 degradation in the vascular wall. Moreover, a series of evidence suggested that ROS plays a crucial role in the development of AD (28). Indeed, our results showed that the ROS production in aortic tissues was significantly reduced when NEU1 was deficient in macrophage. Taken together, our data suggest that NEU1 accelerates the development of AD by enhancing inflammatory response, cell apoptosis, oxidative stress, and ECM degradation.

We further investigated how NEU1 in macrophages promotes inflammation in the local microenvironment. Studies have shown that Ang II promotes the inflammatory response of local tissues by regulating the recruitment and polarization of macrophages, and thus induces the onset of AD in humans and experimental animals (8). We therefore tested whether NEU1 KO in macrophages could also affect the occurrence and development of AD by changing the polarization state of macrophages. Flow cytometry and immunofluorescence staining revealed that macrophage NEU1 KO significantly reduced the M1 macrophages and upregulated the M2 macrophages. The macrophages in the aortic tissues were sorted by flow cytometry and detected by q-PCR, and the results showed that when NEU1 was knocked out, the pro-inflammatory-related (M1-like) gene was significantly downregulated, whereas the anti-inflammatory-related (M2-like) gene was significantly upregulated. In addition, to further confirm these findings, we extracted macrophages from the NEU1^F/F^ mice and their controls. After Ang II treatment, stained with M1 and M2 macrophage markers, it was confirmed that NEU1 promoted the onset of dissection by regulating macrophage into M1 phenotype.

In summary, we showed that NEU1 was upregulated in aortic tissues (especially in CD68^+^ macrophages) from the BAPN-induced AD mouse model. Moreover, the genetic deletion retarded AD progression by directly suppressing the production of pro-inflammatory macrophages. Collectively, these data suggest that NEU1 may potentially serve as a new therapeutic target for AD.

## Data Availability Statement

The raw data supporting the conclusions of this article will be made available by the authors, without undue reservation.

## Ethics Statement

The animal study was reviewed and approved by the Ethics Committee of The First Affiliated Hospital of Nanchang University.

## Author Contributions

QW, ZC, XP, ZZ, AL, and JZ designed the study and revised critically the manuscript. QW, ZC, XP, ZZ, AL, JG, LM, HS, and KY performed experiments. KY and SZ provided materials, performed measurements, and analyzed data. ZZ and KY wrote the manuscript. All authors approved final version of manuscript submitted.

## Funding

This study was supported by National Natural Science Foundation of China (Grant Nos. 81800324, 81960061, and 81600241), Natural Science Foundation of Jiangxi Province (Grant Nos. 20192ACBL21040 and 20204BCJ23017), Key Project of Science and Technology Research Funds of Department of Education of Jiangxi Province (Grant Nos. GJJ170136 and GJJ170005), and Science Project of Department of Health Commission of Jiangxi Province (Grant No. 20203094).

## Conflict of Interest

The authors declare that the research was conducted in the absence of any commercial or financial relationships that could be construed as a potential conflict of interest.

## Publisher's Note

All claims expressed in this article are solely those of the authors and do not necessarily represent those of their affiliated organizations, or those of the publisher, the editors and the reviewers. Any product that may be evaluated in this article, or claim that may be made by its manufacturer, is not guaranteed or endorsed by the publisher.

## References

[1] Vaideeswar P Kundu S Singaravel S Tyagi S. Spontaneous aortic rupture: report of two cases with review of literature. Indian J Pathol Microbiol. (2021) 64:152–4. 10.4103/IJPM.IJPM_382_20 33433428

[2] Yang YY Li LY Jiao XL Jia LX Zhang XP Wang YL. Intermittent hypoxia alleviates beta-aminopropionitrile monofumarate induced thoracic aortic dissection in C57BL/6 mice. Eur J Vasc Endovasc Surg. (2020) 59:1000–10. 10.1016/j.ejvs.2019.10.014 31879145

[3] Santos LCF de Paiva MAF Santana MVL Mendes R Tenorio PP. Could we adopt serum Tenascin-C assays to determine prognosis in aortic aneurysms and dissections?. J Vasc Bras. (2021) 20:e20200165. 10.1590/1677-5449.200165 34456984 PMC8366403

[4] Juraszek A Czerny M Rylski B. Update in aortic dissection. Trends Cardiovasc Med. (2021) 8:8. 10.1016/j.tcm.2021.08.008 34411744

[5] Ohno-Urabe S Aoki H Nishihara M Furusho A Hirakata S Nishida N. Role of macrophage socs3 in the pathogenesis of aortic dissection. J Am Heart Assoc. (2018) 7:389. 10.1161/JAHA.117.007389 29343476 PMC5850160

[6] Nienaber CA Clough RE. Management of acute aortic dissection. Lancet. (2015) 385:800–11. 10.1016/S0140-6736(14)61005-9 25662791

[7] Wu D Choi JC Sameri A Minard CG Coselli JS Shen YH. Inflammatory cell infiltrates in acute and chronic thoracic aortic. Dissection Aorta. (2013) 1:259–67. 10.12945/j.aorta.2013.13-044 26798703 PMC4682718

[8] Wang X Zhang H Ge Y Cao L He Y Sun G. AT1R regulates macrophage polarization through YAP and regulates aortic dissection incidence. Front Physiol. (2021) 12:644903. 10.3389/fphys.2021.644903 34305627 PMC8299470

[9] Muller BT Modlich O Prisack HB Bojar H Schipke JD Goecke T. Gene expression profiles in the acutely dissected human aorta. Eur J Vasc Endovasc Surg. (2002) 24:356–64. 10.1053/ejvs.2002.1731 12323180

[10] Tieu BC Lee C Sun H Lejeune W Recinos A 3rd Ju X. An adventitial IL-6/MCP1 amplification loop accelerates macrophage-mediated vascular inflammation leading to aortic dissection in mice. J Clin Invest. (2009) 119:3637–51. 10.1172/JCI38308 19920349 PMC2786788

[11] Anzai A Shimoda M Endo J Kohno T Katsumata Y Matsuhashi T. Adventitial CXCL1/G-CSF expression in response to acute aortic dissection triggers local neutrophil recruitment and activation leading to aortic rupture. Circ Res. (2015) 116:612–23. 10.1161/CIRCRESAHA.116.304918 25563839

[12] Son BK Sawaki D Tomida S Fujita D Aizawa K Aoki H. Granulocyte macrophage colony-stimulating factor is required for aortic dissection/intramural haematoma. Nat Commun. (2015) 6:6994. 10.1038/ncomms7994 25923510

[13] Ju X Ijaz T Sun H Ray S Lejeune W Lee C. Interleukin-6-signal transducer and activator of transcription-3 signaling mediates aortic dissections induced by angiotensin II via the T-helper lymphocyte 17-interleukin 17 axis in C57BL/6 mice. Arterioscler Thromb Vasc Biol. (2013) 33:1612–21. 10.1161/ATVBAHA.112.301049 23685554 PMC3818154

[14] Kibler WB Goldberg C Chandler TJ. Functional biomechanical deficits in running athletes with plantar fasciitis. Am J Sports Med. (1991) 19:66–71. 10.1097/00042752-199107000-00021 1672577

[15] Liu J Yang Y Liu X Widjaya AS Jiang B Jiang Y. Macrophage-biomimetic anti-inflammatory liposomes for homing and treating of aortic dissection. J Control Release. (2021) 337:224–35. 10.1016/j.jconrel.2021.07.032 34298057

[16] Chen QQ Ma G Liu JF Cai YY Zhang JY Wei TT. Neuraminidase 1 is a driver of experimental cardiac hypertrophy. Eur Heart J. (2021) 2021:ehab347. 10.1093/eurheartj/ehab347 34179969

[17] Seyrantepe V Poupetova H Froissart R Zabot MT Maire I Pshezhetsky AV. Molecular pathology of NEU1 gene in sialidosis. Hum Mutat. (2003) 22:343–52. 10.1002/humu.10268 14517945

[18] Khan A Das S Sergi C. Therapeutic potential of NEU1 in Alzheimer's disease *via* the immune system. Am J Alzheimers Dis Other Demen. (2021) 36:1533317521996147. 10.1177/1533317521996147 33719595 PMC10624071

[19] Abdulkhalek S Amith SR Franchuk SL Jayanth P Guo M Finlay T. NEU1 sialidase and matrix metalloproteinase-9 cross-talk is essential for Toll-like receptor activation and cellular signaling. J Biol Chem. (2011) 286:36532–49. 10.1074/jbc.M111.237578 21873432 PMC3196117

[20] Sieve I Ricke-Hoch M Kasten M Battmer K Stapel B Falk CS. A positive feedback loop between IL-1beta, LPS and NEU1 may promote atherosclerosis by enhancing a pro-inflammatory state in monocytes and macrophages. Vascul Pharmacol. (2018) 103–5:16–28. 10.1016/j.vph.2018.01.005 29371126

[21] Sieve I Munster-Kuhnel AK Hilfiker-Kleiner D. Regulation and function of endothelial glycocalyx layer in vascular diseases. Vascul Pharmacol. (2018) 100:26–33. 10.1016/j.vph.2017.09.002 28919014

[22] Pshezhetsky AV Ashmarina LI. Desialylation of surface receptors as a new dimension in cell signaling. Biochemistry. (2013) 78:736–45. 10.1134/S0006297913070067 24010837

[23] Heimerl M Sieve I Ricke-Hoch M Erschow S Battmer K Scherr M. Neuraminidase-1 promotes heart failure after ischemia/reperfusion injury by affecting cardiomyocytes and invading monocytes/macrophages. Basic Res Cardiol. (2020) 115:62. 10.1007/s00395-020-00821-z 32975669 PMC7519006

[24] Xiao Y Sun Y Ma X Wang C Zhang L Wang J. MicroRNA-22 inhibits the apoptosis of vascular smooth muscle cell by targeting p38MAPKalpha in vascular remodeling of aortic dissection. Mol Ther Nucleic Acids. (2020) 22:1051–62. 10.1016/j.omtn.2020.08.018 33294292 PMC7691156

[25] McMurtry MS Bonnet S Wu X Dyck JR Haromy A Hashimoto K. Dichloroacetate prevents and reverses pulmonary hypertension by inducing pulmonary artery smooth muscle cell apoptosis. Circ Res. (2004) 95:830–40. 10.1161/01.RES.0000145360.16770.9f 15375007

[26] Liao WL Tan MW Yuan Y Wang GK Wang C Tang H. Brahma-related gene 1 inhibits proliferation and migration of human aortic smooth muscle cells by directly up-regulating Ras-related associated with diabetes in the pathophysiologic processes of aortic dissection. J Thorac Cardiovasc Surg. (2015) 150:1292–301 e2. 10.1016/j.jtcvs.2015.08.010 26344687

[27] Zhang J Liu F He YB Zhang W Ma WR Xing J. Polycystin-1 downregulation induced vascular smooth muscle cells phenotypic alteration and extracellular matrix remodeling in thoracic aortic dissection. Front Physiol. (2020) 11:548055. 10.3389/fphys.2020.548055 33071810 PMC7541897

[28] Fan LM Douglas G Bendall JK McNeill E Crabtree MJ Hale AB. Endothelial cell-specific reactive oxygen species production increases susceptibility to aortic dissection. Circulation. (2014) 129:2661–72. 10.1161/CIRCULATIONAHA.113.005062 24807872 PMC5357047

