# Supplementary material for: Neuraminidase 1 Exacerbating Aortic Dissection by Governing a Pro-Inflammatory Program in Macrophages
Source: Front Cardiovasc Med. 2021 Nov 18;8:788645. doi: 10.3389/fcvm.2021.788645 (PMC8639188; doi:10.3389/fcvm.2021.788645)
Supplement: Supplementary file 1 [file Image_1.pdf]

Supplementary material

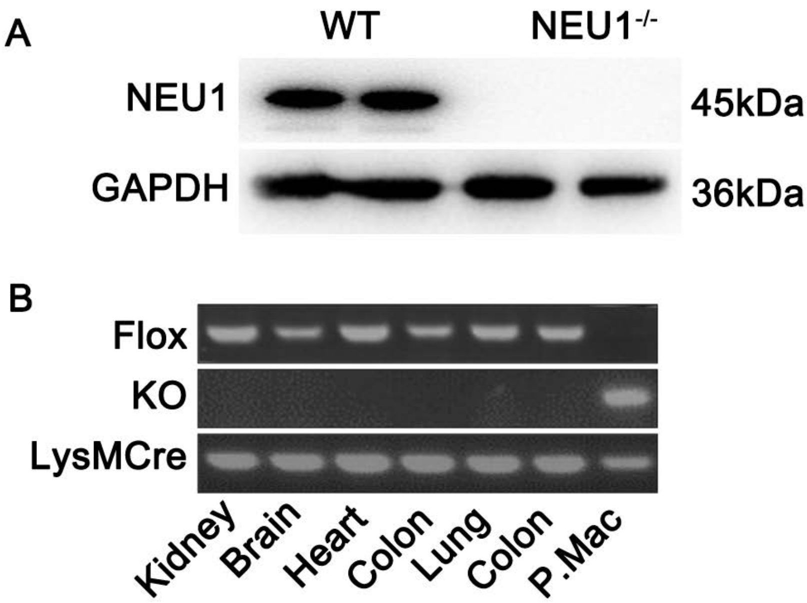

Supplementary Figure1. Characterization of global and macrophage–specific NEU1-deficient mice. (A), Western blot analysis of NEU1 expression in aortic tissues from C57BL/6J and global NEU1 knockout mice. (B), PCR analysis on genomic DNA derived from various organs of $NEU1^{F/F}$; $LysM^{Cre}$ mice (NEU1 CKO).
